# Inequality Measure of Leaf Area Distribution for a Drought-Tolerant Landscape Plant

**DOI:** 10.3390/plants12173143

**Published:** 2023-08-31

**Authors:** Lichao Huang, David A. Ratkowsky, Cang Hui, Johan Gielis, Meng Lian, Weihao Yao, Qiying Li, Liuyue Zhang, Peijian Shi

**Affiliations:** 1 Tourism and Air Service College, Guizhou Minzu University, Guiyang 550025, China; huanglic1002@163.com; 2 Bamboo Research Institute, College of Ecology and Environment, Nanjing Forestry University, Nanjing 210037, China; mlian@njfu.edu.cn (M.L.); whyao@njfu.edu.cn (W.Y.); lqyin@njfu.edu.cn (Q.L.); 3 Tasmanian Institute of Agriculture, University of Tasmania, Hobart 7001, TS, Australia; d.ratkowsky@utas.edu.au; 4 Centre for Invasion Biology, Department of Mathematical Sciences, Stellenbosch University, Stellenbosch 7602, South Africa; chui@sun.ac.za; 5 Mathematical and Physical Biosciences, African Institute for Mathematical Sciences, Cape Town 7945, South Africa; 6 Department of Biosciences Engineering, University of Antwerp, B-2020 Antwerp, Belgium; johan.gielis@uantwerpen.be; 7 Key Laboratory of Bio-Resource and Eco-Environment of Ministry of Education, College of Life Sciences, Sichuan University, Chengdu 610064, China; zhang_liuyue@163.com

**Keywords:** coefficient of variation, Gini index, landscape plant, Lorenz curve, Theil index

## Abstract

Measuring the inequality of leaf area distribution per plant (ILAD) can provide a useful tool for quantifying the influences of intra- and interspecific competition, foraging behavior of herbivores, and environmental stress on plants’ above-ground architectural structures and survival strategies. Despite its importance, there has been limited research on this issue. This paper aims to fill this gap by comparing four inequality indices to measure ILAD, using indices for quantifying household income that are commonly used in economics, including the Gini index (which is based on the Lorenz curve), the coefficient of variation, the Theil index, and the mean log deviation index. We measured the area of all leaves for 240 individual plants of the species *Shibataea chinensis* Nakai, a drought-tolerant landscape plant found in southern China. A three-parameter performance equation was fitted to observations of the cumulative proportion of leaf area vs. the cumulative proportion of leaves per plant to calculate the Gini index for each individual specimen of *S. chinensis*. The performance equation was demonstrated to be valid in describing the rotated and right shifted Lorenz curve, given that >96% of root-mean-square error values were smaller than 0.004 for 240 individual plants. By examining the correlation between any of the six possible pairs of indices among the Gini index, the coefficient of variation, the Theil index, and the mean log deviation index, the data show that these indices are closely related and can be used interchangeably to quantify ILAD.

## 1. Introduction

As primary organs of photosynthesis, leaves play a critical role in capturing light and absorbing carbon dioxide (CO_2_) from the atmosphere before converting them into bioenergy [1]. However, leaf area, as an important plant functional trait, is influenced by a myriad of factors [2]. For example, the climate can impact the leaf morphology of grasses, and cause them to have shorter and narrower leaves under cold or dry conditions [3]. Additionally, the above-ground architectural structures of plants can affect leaf area distribution, e.g., most woody species can be regarded as modular organisms, and their branching patterns are repetitively expressed during the growth of shoots which produce a ‘modular’ or ‘metameric’ architecture that is regulated by genetic factors [4]. Leaves are distributed on the ‘modular’ branches. Light distribution plays a significant role in the expansion of leaf area through trophic and light quality effects [5]. The neighboring plants can also affect branch morphology, with higher plants responding to the proximity of other plants with plastic morphological and physiological changes, including variations in branching rate, accelerated stem elongation, and stem bending away from adjacent plants [6]. As a response to the above-mentioned influencing factors, plants allocate their biomass differently among individual leaves at different longitudinal layers aboveground. For instance, *Ficus pandurata* Hance has large leaves because water and nutrients can efficiently be transported between leaves and stems owing to its simple branching pattern. In contrast, *Portulacaria afra* Jacq. has small leaves, because large leaves can increase the cost of transporting assimilates in order to maintain mechanical stability due to its complex branching pattern. When the total biomass investment into leaves is fixed, there should be an optimal allocation of biomass to different leaves per plant to maximize photosynthetic benefits. Accurately measuring the degree of inequality in leaf area distribution per plant (ILAD) can gauge the influences of many abiotic and biotic factors, e.g., plant spatial density, light distribution, branching patterns, and the foraging behavior of herbivores on the above-ground architecture of plants. Thus, it is meaningful to quantify the degree of ILAD. However, we know of no published study that has focused on quantifying this measure.

The Lorenz curve, widely used in economics, visually describes the degree of income inequality among all individual households in a country or an economy. It ranks the household income from small to large, with the cumulative proportion of households as the horizontal axis and the cumulative proportion of household income as the vertical axis. This curve starts at the origin (0, 0) and ends at the coordinates (1, 1) in the Cartesian coordinate system, with a straight line through the two points denoting an absolute equality of income distribution. As this monotonically increasing curve moves further away from a straight line (i.e., the line of absolute equality), a greater level of income inequality among households is signified, indicating a higher concentration of wealth among a smaller proportion of individuals. When comparing two different economies, if the Lorenz curve of the former economy is located above that of the latter, the degree of income inequality in the former economy is lower than in the latter. When two Lorenz curves intersect, it is difficult to visually determine the difference of income inequality between the two economies. In that case, an indicator, referred to as the Gini index [7], was proposed to quantify income inequality by calculating the ratio of the area between the Lorenz curve and the egalitarian line to the area below the egalitarian line. There are many mathematical equations that can be used to describe the Lorenz curve [8,9,10,11,12,13,14,15,16,17,18,19]. There is also the Theil index of inequality [20,21] which can be partitioned into inter-group components and intra-group components [22]. This approach allows for a more nuanced understanding of the factors driving inequality.

Photosynthesis is at the basis of life, and photosynthetic organisms have developed a variety of forms and shapes to capture sunlight and absorb CO_2_, both in their constructional organization and the shape of their photosynthetic organs. Plants have developed, in response to environmental challenges, a variety of growth forms ranging from herbs, annuals, shrubs, and trees, to the metamorphosis of stems and leaves present in succulent forms, or the plagiotropically growing rhizomes in grasses and bamboos. The constructional organization is that of an iteration of metamers or phytomers, with constructional units consisting of a part of the stem, comprising a node and the leaf at that node, with a bud subtended at that node if present. The leaves can take on many forms, with foliage leaves being the main photosynthetic organs of plants. They can be connected directly to the node, or via a petiole. In the case of grasses and bamboos, the culm sheath connects to the node, and via a shoulder (e.g., in corn) or via a pseudo-petiole (in bamboos), the foliage leaf is connected to the culm sheath. A major question is the variation in the size and form of foliage leaves within one plant. A problem with the application of the Gini index to quantify ILAD is that it is difficult to accurately measure the total leaf area for a tree with a large number of leaves, and it is also difficult to do so for herbs because of the lack of leaf petioles. Fortunately, Bambusoideae (a subfamily of Poaceae) is a group of plants that are of intermediate form between herbaceous and woody plants, comprising 116 genera and approximately 1439 species [23]. Among this group of plants, there are some herbaceous dwarf bamboo species (e.g., *Shibataea chinensis* Nakai) which have relatively fewer leaves than woody bamboo species (e.g., *Phyllostachys edulis* (Carriere) J. Houzeau), making it convenient to measure the degree of ILAD. *S. chinensis* is widely distributed in southern China, and has been widely planted in urban parks, especially northern parks, as a drought-tolerant landscape plant to please tourists and play an important role in carbon sequestration [24,25,26]. In the present study, the Gini index from the Lorenz curve, the coefficient of variation, the Theil index, and the mean log deviation index were used to quantify the degree of ILAD in *S. chinensis*, which can potentially gauge the influences of the intra- and interspecific competition, the foraging behavior in herbivores, and the environmental stress on plants’ above-ground architectural structures and survival strategies.

## 2. Materials and Methods

### 2.1. Leaf Sampling Information

**Figure 1:**
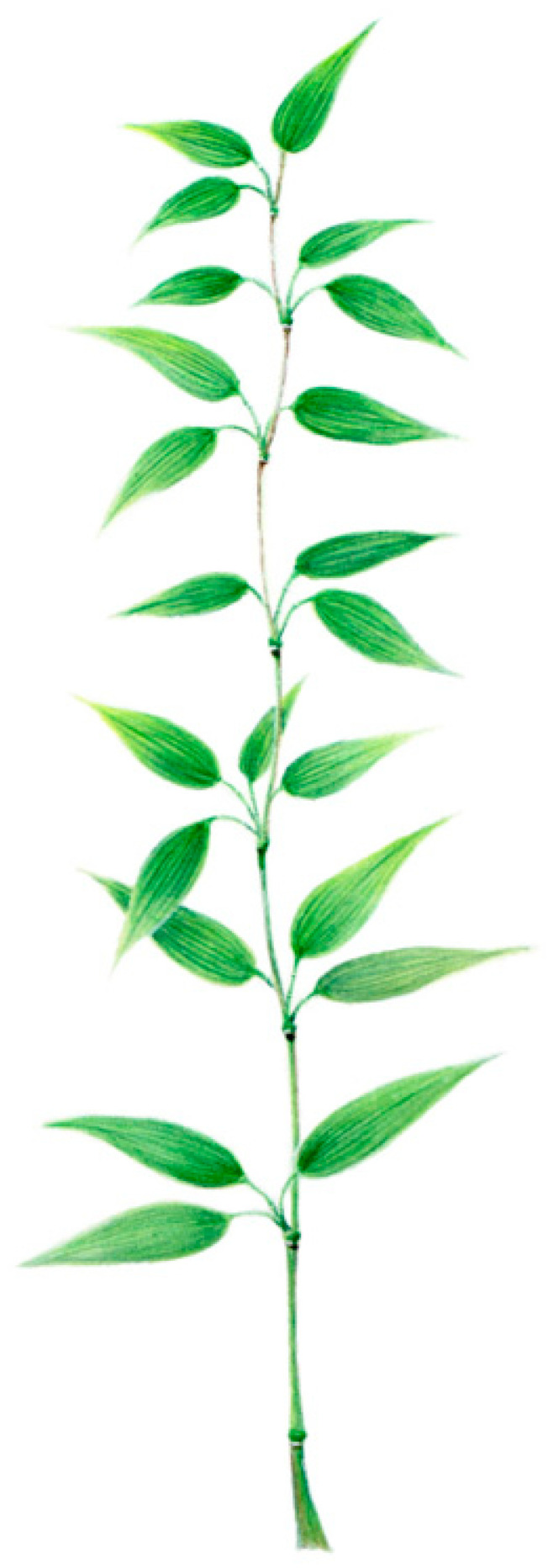
Freehand drawing of the above-ground part of *S. chinensis*.

We randomly sampled 240 *S. chinensis* plants, a scattered bamboo species, growing in the Nanjing Forestry University campus (118°48′53″ E, 32°4′52″ N), in October 2023. The number of leaves per plant did not exceed 40. Figure 1 shows the above-ground architectural structure of this dwarf bamboo species. We cut down each culm at the ground, wrapped the samples with wet paper, and took them back to the lab within one hour. The leaves were cut from the plant, and the pseudo-petioles were removed.

### 2.2. Data Acquisition

Each leaf was scanned as a .jpg image with a photo scanner (V550, Epson Indonesia, Batam, Indonesia). We used Adobe Photoshop (version 13.0; Adobe, San Jose, CA, USA) to convert it into a black and white .bmp image. This procedure is based on Matlab (version ≥ 2009a; MathWorks, Natick, MA, USA) and proposed by refs. [27,28] for the extraction of leaf boundary coordinate data from the black and white image. Leaf area was calculated using the ‘bilat’ function of the ‘biogeom’ package (version 1.3.5) based on the statistical software R (version 4.2.0) [29,30]. The raw data regarding the leaf size of all 240 *S. chinensis* plants are accessible from the online Supplementary Table S1.

### 2.3. Fitting the Lorenz Curve

**Figure 2:**
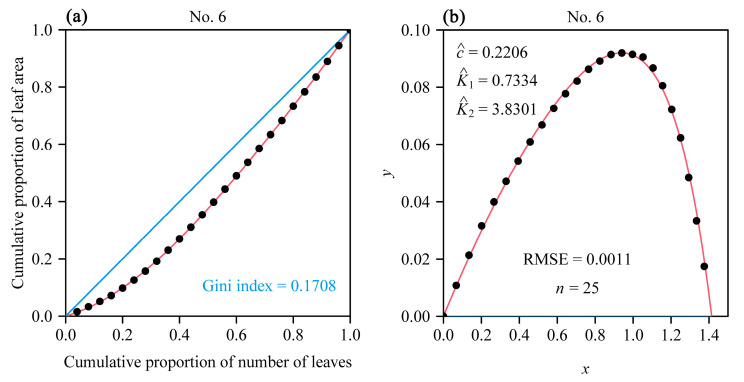
The fitted results for an individual plant (No. 6 in this example) of *S. chinensis* using the performance equation. Panel (**a**) shows the comparison of the observations and the predicted original Lorenz curve, and the blue 45° straight line represents the line of absolute equality; panel (**b**) shows the comparison of the observations and the predicted Lorenz curve after being rotated and right shifted. The closed circles represent the observations, and the red curve represents predicted values. In panel (**b**), the letters *c*, *K*_1_, and *K*_2_ with hats represent the estimated values of parameters for the performance equation; RMSE represents root-mean-square error; *n* represents the number of leaves on this individual plant. The Gini index is estimated as double the area under the performance equation (the red curve in panel (**b**)).

The performance equation was proposed to fit the Lorenz curve [19,31,32], which required the coordinates of the cumulative proportion of leaf area (vertical) vs. the cumulative proportion of leaves (horizontal) in the original Cartesian space to be rotated counterclockwise by 135°, and then the new coordinates shifted to the right by a distance of $\sqrt{2}$ (see Figure 2):

(1) $$y=c\left(1-e^{-K_{1}x}\right)\left(1-e^{K_{2}(x-\sqrt{2})}\right)$$

where *y* and *x* are the new coordinates after the rotation and right shift of the original Lorenz curve.

The nonlinear least squares method based on the Nelder–Mead algorithm [33] was used to estimate the three parameters by minimizing the residual sum of squares (RSS) between the observed and predicted *y* values. We used the root-mean-square error (RMSE) to reflect the goodness of fit:

(2) $$\mathrm{RMSE}=\sqrt{\mathrm{RSS}/n}$$

where *n* represents the number of leaves on a plant.

### 2.4. Four Indices for Quantifying ILAD

We used four indices to reflect ILAD, comprising the Gini index, the coefficient of variation, the Theil index, and the mean log deviation index.

(i) The Gini index is calculated as follows:

(3) $$\mathrm{Gini}\;\mathrm{index}=2\times \int_{0}^{\sqrt{2}}ydx$$

where *y* is given by Equation (1).

(ii) The coefficient of variation (CV) is the ratio of the standard error (SE) to the mean leaf area (μ) per plant, which equals:

(4) $$\mathrm{CV}=\frac{\mathrm{SE}}{\mu }$$

There is a need to note that CV is usually presented in percentage form. However, we did not use its percentage form as it would be less convenient in the present work.

(iii) The Theil index [20,34] also uses the concept of relative entropy in information theory to calculate income inequality:

(5) $$T_{1}=\frac{1}{n}\sum_{i=1}^{n}\frac{A_{i}}{\mu }\log\left(\frac{A_{i}}{\mu }\right)$$

where *n* represents the number of leaves on the individual plant, *A_i_* represents the area of the *i*-th leaf of the individual plant, and μ represents the average leaf area of the individual plant.

(iv) The mean log deviation index uses the concept of relative entropy as well, defined as [20,34]:

(6) $$T_{2}=-\frac{1}{n}\sum_{i=1}^{n}\log\left(\frac{A_{i}}{\mu }\right)$$

where *n*, *A_i_*, and μ are defined as in (iii) above.

The calculation of the above four indices was carried out using the statistical software R (version 4.2.0) [30].

## 3. Results

**Figure 3:**
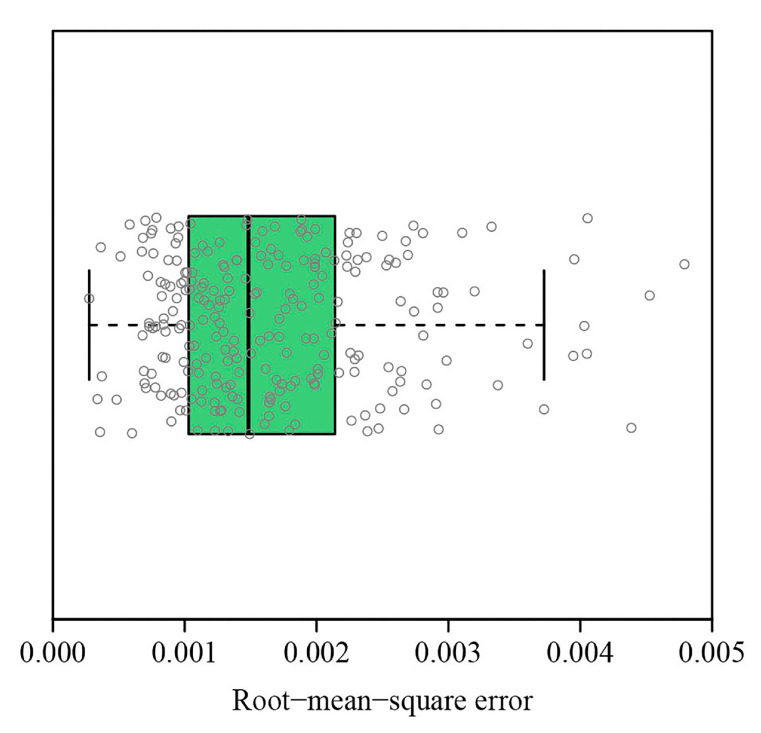
Boxplot of the root-mean-square errors observed when fitting the rotated and right shifted data, which indicate the cumulative proportion of leaf area vs. the cumulative proportion of leaves per plant, by using the performance equation for the 240 individual plants.

Most RMSE values (>96%) obtained using the performance equation were smaller than 0.004, which demonstrated the validity of the performance equation in fitting the coordinates, after rotating and right shifting, of the cumulative proportion of leaf area vs. the cumulative proportion of leaves per plant (Figure 3). Figure 2 shows the fitted results of 1 randomly selected plant from among the 240 individual plants. The fitted results for the remaining 239 individual plants can be accessed in the online Table S2.

**Figure 4:**
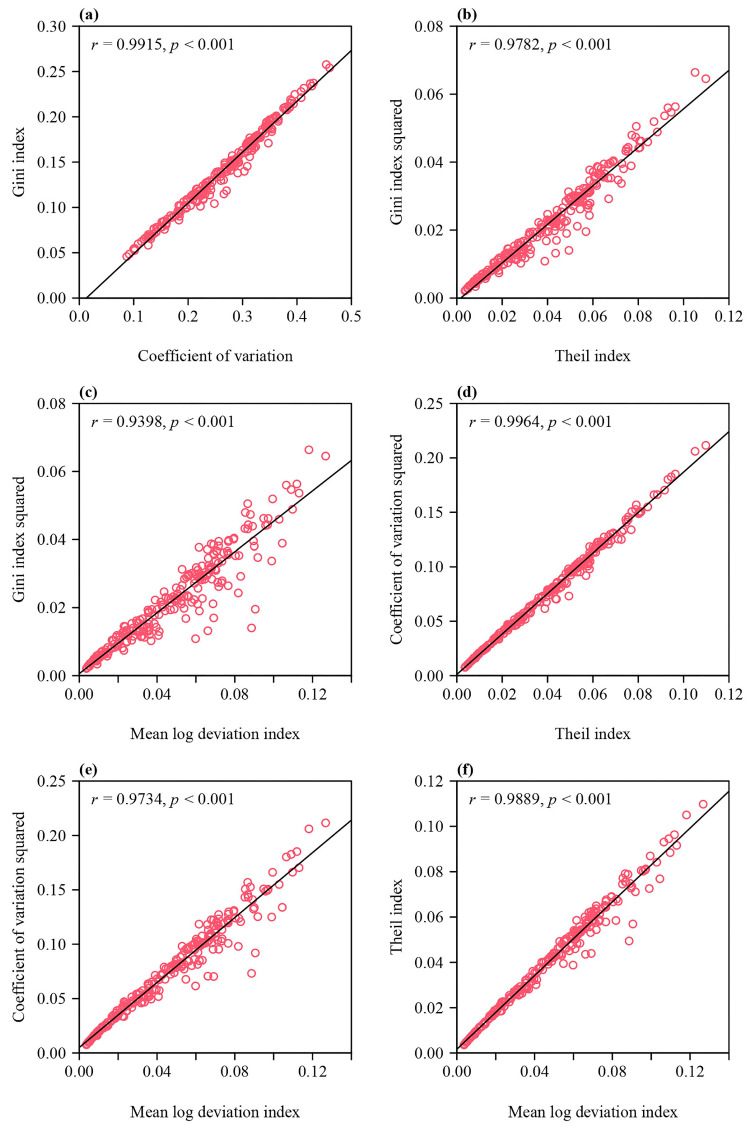
Correlations between pairs of indices for quantifying the ILAD. (**a**) The Gini index vs. the coefficient of variation, (**b**) the Gini index squared vs. the Theil index, (**c**) the Gini index squared vs. the mean log deviation index, (**d**) the coefficient of variation squared vs. the Theil index, (**e**) the coefficient of variation squared vs. the mean log deviation index, and (**f**) the Theil index vs. the mean log deviation index. Here, *r* represents the correlation coefficient; *p* is the *p*-value of the correlation test; the small open circles represent 240 individual plants; and the straight line is the regression line.

Based on the fitted results using the performance equation, the Gini index for all 240 *S. chinensis* plants had a mean of 0.1413 with a standard error of 0.0468; the coefficient of variation was also calculated, with a mean of 0.2649 and a standard error of 0.0827; the Theil index had a mean of 0.0408 with a standard error of 0.0232; and the mean log deviation index had a mean of 0.0482 and a standard error of 0.0282 (see the online Table S3). Pairs of indices among the Gini index (or Gini index squared), the coefficient of variation (or coefficient of variation squared), the Theil index, and the mean log deviation index were significantly correlated (*p* < 0.01; Figure 4), indicating that these indices for measuring ILAD can substitute for one another.

**Figure 5:**
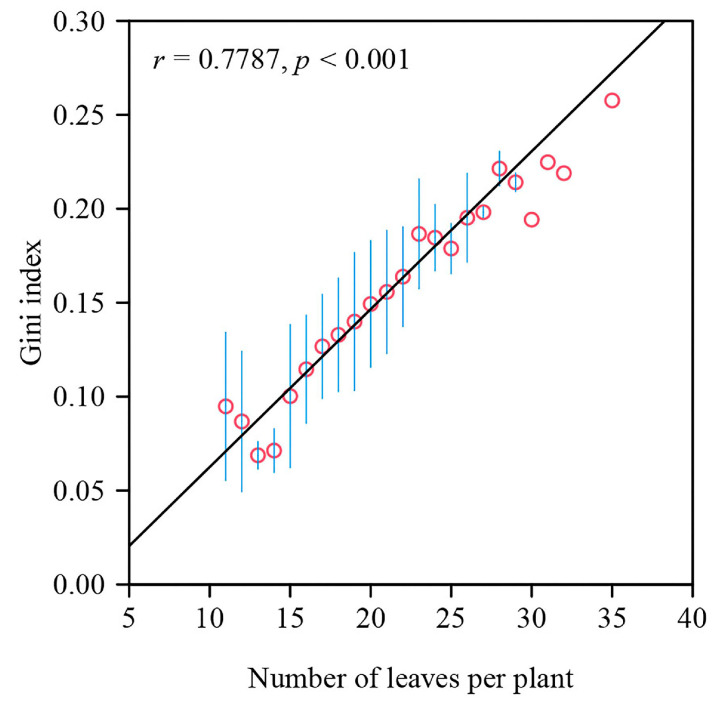
Correlation between the Gini index of the leaf area distribution per plant and the number of leaves per plant. The small red open circles represent the means of the observations of Gini indices for a particular number of leaves, and the blue vertical bars represent the standard errors of the Gini indices (when only 1 of the 240 plants had that particular number of leaves, no vertical bar is shown). Here, *r* represents the correlation coefficient; *p* is the *p*-value of the correlation test; and the straight line is the regression line.

Figure 5 shows a significant positive correlation (*p* < 0.05) between the Gini index and the number of leaves per specimen of *S. chinensis*. Since the Gini index is a measure of ILAD, this indicates that the larger the number of leaves per plant, the less equal the leaf area distribution.

## 4. Discussion

In this section, we discuss the aspects that contribute to the robust correlation between pairs of values of the four indices measuring ILAD, identify potential ecological applications for the four indices, and present the strengths and drawbacks of the four indices. Additionally, we also discuss the relationship between the Gini index of leaf area distribution and the number of leaves per plant.

### 4.1. The Links among Four Indices of Measuring ILAD

The generalized entropy index (GEI) utilizes the concept of entropy in information theory, along with the concept of average information content, and is one of the most important indicators to measure the degree of income distribution inequality in an economy [34,35].

(7) $$\mathrm{GEI}\left(\alpha \right)=\left\{\begin{array}{ll} \frac{1}{n}\frac{1}{\alpha (\alpha -1)}\sum_{i=1}^{n}\left[{\left(\frac{A_{i}}{\mu }\right)}^{\alpha }-1\right] & \alpha \neq 0,\;1\\ \frac{1}{n}\sum_{i=1}^{n}\frac{A_{i}}{\mu }\log\left(\frac{A_{i}}{\mu }\right) & \alpha =1\\ -\frac{1}{n}\sum_{i=1}^{n}\log\left(\frac{A_{i}}{\mu }\right) & \alpha =0 \end{array}\right.$$

where α is a non-negative parameter; the larger the parameter α, the greater the generalized entropy index’s sensitivity to differences at the top range of the distribution, while the smaller the parameter α, the greater its sensitivity to low-end differences. The expression of GEI at α = 1 is an asymptotic expression when α→1. The GEI has many good properties, such as symmetry, simple additivity, decomposability, and proportion invariance.

The Theil index is the generalized entropy index with α = 1, and the mean log deviation index is the generalized entropy index with α = 0. Half of CV^2^ is the generalized entropy index with α = 2:

(8) $$\mathrm{GEI}\left(\alpha =2\right)=\frac{1}{2}\left[\frac{1}{n}\sum_{i=1}^{n}{\left(\frac{A_{i}}{\mu }\right)}^{2}-1\right]=\frac{1}{2}\left[\frac{1}{n}\sum_{i=1}^{n}{\frac{\left(A_{i}-\mu \right)}{\mu^{2}}}^{2}\right]=\frac{1}{2}{\mathrm{CV}}^{2}$$

Rohde [35] demonstrated a link between the generalized entropy and the Lorenz curve, and concluded that the Lorenz curve is the foundation of most inequality measures, including the generalized entropy. The present study provides support for this conclusion based on empirical data (see Figure 4).

The Theil index is a great tool for regional inequality measure. Due to its excellent decomposability inherited from the generalized entropy index, it performs well in measuring the degree of inequality at different levels and their contribution to overall inequality. Based on the number of stages decomposed, it can be divided into two indices: the one-stage Theil index, and the multi-stage Theil index. The one-stage Theil decomposition method decomposes the overall regional variation into within-region and between-region, and calculates the contribution of different parts to the overall differences. The multi-stage Theil index can further decompose within-region inequality, e.g., the two-stage Theil index can decompose within-region inequality into intraprovincial and interprovincial [22]. Relatively speaking, the calculation of CV is simpler, but the Lorenz curve can visually generate an unbalanced intuitive feeling. The Theil index has excellent decomposability and can be applied to the leaf area observations. For leaves with different layers in height, it can calculate the inter-layer and intra-layer differences and their contributions to the total differences. Users can choose among these three indicators according to different needs.

### 4.2. Applications of Four Indices to Plant Ecology

The inequality of leaf area distribution per plant (ILAD) can help to explain the strategy of resource allocation among different plant organs, and the measure of ILAD can be used to assess the efficiency of resource distribution and utilization by plants. In fact, the inequality measures for plant size distribution and seed size distribution have been used to assess the influence of stress factors like intra- or inter-specific competition on plant growth [36,37,38]. Similarly, ILAD can be used as an indicator of plants’ functional traits and fitness, as it reflects the plants’ ability to allocate resources efficiently. When using the mean leaf area as a predictor variable in a model to evaluate its influence on a special dependent variable, such as the yield of an individual plant, the coefficient of variation (CV) in leaf area distribution becomes feasible to assess its representativeness on the overall leaf area. The Theil index’s exceptional decomposability enables the variation of leaf area into intra-layer (e.g., for the pericycle order) and inter-layer variation, and also provides a means of quantifying the respective contributions of intra- and inter-layer parts to the total variation. If there is a small variation within a layer, the leaf area on the same layer can be considered to be uniform (similar to the analysis of variance), thereby simplifying the calculation of leaf size distribution on different layers of a plant.

From a larger perspective, by measuring the physiological characteristics of plants, especially those produced as a response to environment stress, e.g., CO_2_ elevation, the corresponding CV in a physiological characteristic of interest can be calculated to evaluate the degree of variation in this characteristic across different plants, and to understand the adaptability and stability of plants. The Theil index (or the Gini index) can be calculated by using the number and composition of different species in a plant community to assess the degree of species diversity in the community. The Theil index (or the Gini index) can also be used to assess the risk of biological invasion and the stability of ecosystems. In ecology, if a region has a large Theil index, this indicates that the size distribution of species is uneven, making it susceptible to invasion by alien invasive species. If a region has a small Theil index, this indicates a uniform distribution of species, and the stability of the ecosystem is high [39,40]. The Theil index can also be used as an indicator for formulating conservation strategies of biodiversity. By calculating the Theil index, the composition and spatial distribution of species can be analyzed, and the ecological relationships and evolution laws of ecosystems among species can be studied.

### 4.3. Correlation between the Gini Index and Number of Leaves per Plant

The positive correlation between the Gini index and the number of leaves per plant might result from the above-ground architectural structure and light competition [4,41]. The distribution of leaf area is determined by the arrangement and position of leaves on a plant. When there are more leaves on an individual plant in a population, there is a greater possibility for light competition with adjacent plants [42], which tends to lead some leaves to be shaded and bilaterally asymmetrical [43]. Additionally, larger leaves typically require more resources such as water and nutrients, so plants may allocate these resources unequally among leaves in order to maximize overall growth and survival, and the leaf size is correlated with the position of leaves on a plant. These factors contribute to an uneven distribution of leaf area (a higher Gini index) on a plant with a large number of leaves. The above-ground architectural structure and leaf area distribution of plants are also the trade-off result of the available light resources, nutrient transportation costs, and leaf growth and maintenance costs. Therefore, when comparing the Gini indices of different individual plants, it is better to keep balance in individual-to-individual sample sizes in order to eliminate the influence of the difference in sample size across different individual plants.

## 5. Conclusions

We used the three-parameter performance equation to fit our observations of the cumulative proportion of leaf area vs. the cumulative proportion of leaves per plant after being rotated and right shifted, in order to calculate the Gini index to quantify the inequality of leaf area distribution per plant (ILAD), using 240 individual specimens of *Shibataea chinensis* Nakai, a drought-tolerant ecological landscape plant found in southern China. The performance equation was demonstrated to be valid in describing the rotated and right shifted Lorenz curve. Furthermore, we found a significant correlation between pairs of the following four indices: the Gini index, the coefficient of variation, the Theil index, and the mean log deviation index, which were all used to quantify ILAD. We showed that apart from the Gini index, the remaining three indices were actually special cases of the generalized entropy index. In addition, we found a significant positive correlation between the Gini index and the number of leaves per plant (i.e., the sample size for fitting the Lorenz curve). Finally, we discussed potential ecological applications for these indices. The present work provides a useful tool for quantifying ILAD, which can gauge the influence of competition and environmental factors on the above-ground architectural structure of plants, thereby helping design the landscape layout of plants in ecological parks and tourist attractions.

## Data Availability

The data can be found in the online Supplementary Tables S1−S3.

## Supplementary Materials

The following supporting information can be downloaded at: https://www.mdpi.com/article/10.3390/plants12173143/s1, Table S1: Data surrounding the leaf area distributions of 240 individual plants; Table S2: Fitted results of the performance equation; Table S3: Four indices used to measure the inequality of leaf area distribution.
